# Exploring the Potential of Optical Genome Mapping in the Diagnosis and Prognosis of Soft Tissue and Bone Tumors

**DOI:** 10.3390/ijms26062820

**Published:** 2025-03-20

**Authors:** Alejandro Berenguer-Rubio, Esperanza Such, Neus Torres Hernández, Paula González-Rojo, Álvaro Díaz-González, Gayane Avetisyan, Carolina Gil-Aparicio, Judith González-López, Nicolay Pantoja-Borja, Luis Alberto Rubio-Martínez, Soraya Hernández-Girón, María Soledad Valera-Cuesta, Cristina Ramírez-Fuentes, María Simonet-Redondo, Roberto Díaz-Beveridge, Carolina de la Calva, José Vicente Amaya-Valero, Cristina Ballester-Ibáñez, Alessandro Liquori, Francisco Giner, Empar Mayordomo-Aranda

**Affiliations:** 1 Cytogenetic Laboratory, Instituto de Investigación Sanitaria La Fe, 46026 València, Spain; aberrub@upv.edu.es (A.B.-R.); neus_torres@iislafe.es (N.T.H.); diaz_alv@gva.es (Á.D.-G.); gayane_avetisyan@iislafe.es (G.A.); gil_carapa@gva.es (C.G.-A.); 2 Department of Hematology, Hospital Universitari i Politècnic La Fe, 46026 València, Spain; alessandro_liquori@iislafe.es; 3 Centro de Investigación Biomédica en Red de Cáncer (CIBERONC), 28029 Madrid, Spain; 4 Pathology Department, Hospital Universitari i Politècnic La Fe, 46026 València, Spain; paula_gonzalez@iislafe.es (P.G.-R.); gonzalez_judlop@gva.es (J.G.-L.); pantoja_nic@gva.es (N.P.-B.); rubio_lui@gva.es (L.A.R.-M.); hernandez_sor@gva.es (S.H.-G.); valera_sol@gva.es (M.S.V.-C.); franscisco.giner@uv.es (F.G.); mayordomo_emp@gva.es (E.M.-A.); 5 Radiology Department, Hospital Universitari i Politècnic La Fe, 46026 València, Spain; ramirez_crifue@gva.es (C.R.-F.); simonet_mon@gva.es (M.S.-R.); 6 Medical Oncology Service, Hospital Universitari i Politècnic La Fe, 46026 València, Spain; diaz_rob@gva.es; 7 Orthopaedics and Traumatology Department, Hospital Universitari i Politècnic La Fe, 46026 València, Spain; delacalva_car@gva.es (C.d.l.C.); amaya_jos@gva.es (J.V.A.-V.); 8 Department of Orthopaedic Surgery, Hospital Universitari i Politècnic La Fe, 46026 València, Spain; ballester_criiba@gva.es

**Keywords:** soft tissue tumors, bone tumors, optical genome mapping, cytogenetics, diagnosis

## Abstract

Sarcomas are rare malignant tumors of mesenchymal origin with a high misdiagnosis rate due to their heterogeneity and low incidence. Conventional diagnostic techniques, such as Fluorescence In Situ Hybridization (FISH) and Next-Generation Sequencing (NGS), have limitations in detecting structural variations (SVs), copy number variations (CNVs), and predicting clinical behavior. Optical genome mapping (OGM) provides high-resolution genome-wide analysis, improving sarcoma diagnosis and prognosis assessment. This study analyzed 53 sarcoma samples using OGM. Ultra-high molecular weight (UHMW) DNA was extracted from core and resection biopsies, and data acquisition was performed with the *Bionano Saphyr* platform. Bioinformatic pipelines identified structural variations, comparing them with known alterations for each sarcoma subtype. OGM successfully analyzed 62.3% of samples. Diagnostic-defining alterations were found in 95.2% of cases, refining diagnoses and revealing novel oncogenic and tumor suppressor gene alterations. The challenges included DNA extraction and quality issues from some tissue samples. Despite these limitations, OGM proved to be a powerful diagnostic and predictive tool for bone and soft tissue sarcomas, surpassing conventional methods in resolution and scope, enhancing the understanding of sarcoma genetics, and enabling better patient stratification and personalized therapies.

## 1. Introduction

Sarcomas are a rare type of malignant neoplasm arising from mesenchymal tissues, affecting both soft tissue and bone [1,2,3,4,5]. The existing literature reports a misdiagnosis rate of 20–30% [6], which significantly impacts treatment quality and patient prognosis. The difficulty in diagnosing these tumors originates from their low incidence and heterogeneity, as they comprise over 100 subtypes with overlapping histological characteristics, making their study and classification particularly challenging. From a cytogenetic point of view, they can be divided into two large groups: those with simple gene alterations and those with complex karyotypes with multiple structural variations (SVs) and copy number variations (CNVs) without defining alterations [7,8,9]. However, this simplification in classification does not help to predict the clinical behavior, response to treatment, or prognosis of these tumors. This suggests that better characterization of cytogenetic alterations could have a major impact on patient stratification and prognosis.

Concerning sarcoma diagnosis, a biopsy is required for histological, phenotypic, and molecular analysis, which may be performed using various techniques, including percutaneous core needle biopsy. The histological study includes the morphological analysis of the tissue, which allows the determination of the tumor grade based on the study of the parameters described by the ‘Fédération Nationale des Centres de Lutte Contre le Cancer’ (FNCLCC) [10,11]. In addition, immunohistochemical studies help to determine tumor differentiation but not its grade or aggressiveness [10,11].

Molecular studies are increasingly becoming the standard of care in sarcoma diagnosis. However, they are currently performed only when a specific histological diagnosis suggests a simple genetic alteration, such as a characteristic translocation or amplification, particularly in cases of diagnostic uncertainty, unusual clinicopathological presentation, or potential prognostic relevance [8,11]. Fluorescence In Situ Hybridization (FISH) and Next-Generation Sequencing (NGS) panels are widely used molecular techniques with high sensitivity and specificity, but both are inherently targeted. FISH relies on specific probes for predefined genes, restricting its use to known alterations and limiting the ability to analyze all possible genetic changes in the tumor [8,12]. Similarly, NGS panels, despite being fast and cost-effective tools for detecting point mutations, gene fusions, and small genetic abnormalities, require prior knowledge of fusion breakpoints [10,13,14]. Additionally, conventional karyotyping and array comparative genomic hybridization (aCGH) lack diagnostic value [12].

Optical genome mapping (OGM) is a high-resolution cytogenetic technique that uses ultra-high molecular weight DNA (UHMW DNA) (>150 Kbp) to detect SVs (500 bp-1 Mbp), CNVs (>5 Mbp), and complex rearrangements with a Variant Allele Frequency (VAF) higher than 5–10% and with 100–20,000 times more accuracy than traditional karyotyping [15]. UHMW DNA molecules are enzymatically labeled by an enzyme that recognizes a six bp sequence (CTTAAG) present 14 to 17 times per 100 Kbp and then pass through nanochannels where images are taken. The images obtained are converted into digital molecules and assembled bioinformatically to compare the label pattern of the molecules with a reference genome [16]. The overall result is a high-resolution genome-wide analysis that, in a single assay, equals or exceeds the diagnostic scope of multiple combined techniques currently used in clinical cytogenetics.

To date, the use of OGM has been explored mainly in the area of oncohematological diseases, demonstrating that it helps in more accurate diagnosis, better risk stratification, and, thus, more precise treatment [17,18,19,20]. Recently, OGM has started to be used as a diagnostic tool for solid tumors [21,22]. However, only one study has been described where OGM is performed in soft tissue and bone sarcomas, and it was able to detect alterations in 91% of the assessable samples which carried SVs or CNVs [23].

Therefore, the application of OGM could improve the diagnostic classification of sarcomas, their prognosis, and the use or development of targeted therapies, as it could contribute to a better understanding of the molecular mechanisms of these neoplasms. This study aims to compare the findings from the analysis of 53 patients diagnosed with soft tissue or bone sarcomas using OGM, focusing on the expected alterations for each sarcoma subtype and the feasibility of using this technique in routine diagnosis.

## 2. Results

### 2.1. General Data

The age of the patients included in the analysis ranged from 18 to 93 years, with a mean of 59 years. Of the 53 samples included in this study, 33 were from male patients (62.3%) and 20 from female patients (37.7%) (Table 1).

Table 2 provides information regarding tumor groups and sarcoma subtypes included in this study. Regarding the histological nature of the 53 initial tumors, 21 were adipocytic (39.6%), 15 were non-adipocytic (28.3%), 6 were bone tumors (11.3%), 7 were of uncertain differentiation (13.2%), and 4 were small round cell sarcomas (7.6%).

### 2.2. Optical Genome Mapping Analysis

#### 2.2.1. Performance of the Technique

**Figure 1:**
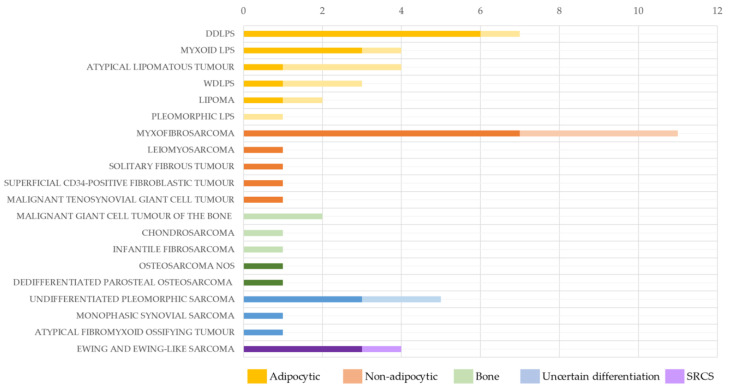
Performance of OGM in the 53 tumors analyzed according to sarcoma subtype. Dark colors show the samples evaluable for OGM, while the light colors show the total number of samples analyzed. DDLPS: dedifferentiated liposarcoma; GC: giant cell; LPS: liposarcoma; NOS: not otherwise specified; SRCS: small round cell sarcomas; WDLPS: well-differentiated liposarcoma.

Of the 53 samples, 33 (62.3%) were evaluable for OGM analysis, with the technique’s effectiveness varying based on the type of starting tissue. High evaluability rates were observed in non-adipocytic samples (11/15, 73.3%), sarcomas of uncertain differentiation (5/7, 71.4%), and small round cell sarcomas (SRCSs) (3/4, 75.0%). In contrast, lower evaluability rates were found in adipose tissue samples (12/21, 57.1%) and bone samples (2/6, 33.3%). Additionally, non-adipocytic tumors with a high extracellular matrix and low cellularity, such as myxofibrosarcomas, also showed reduced evaluability (7/11, 63.6%) (Figure 1).

However, the underlying causes of these limitations varied by tissue type: in adipocytic and myxofibrosarcoma samples, the challenge was primarily related to the difficulty of extracting UHMW DNA, whereas, in bone samples, the main issue was obtaining an assessable OGM analysis.

From now on, only the results of samples considered evaluable by OGM analysis according to the criteria set out in the section on materials and methods are reported.

#### 2.2.2. Quality Parameters

Regarding the quality parameters, 24 of the 33 (72.7%) samples showed good quality with respect to the established values of total DNA collected (>1500 Gbp), map rate (>70%), and coverage (>300×). The average DNA collected was 1435 Gbp (standard deviation: 518.0), average map rate was 72% (standard deviation: 14.2), and average coverage was 328× (standard deviation: 130.8). In addition, nine samples obtained values below the recommended thresholds for some parameters but were still evaluable.

In addition, case 28, corresponding to a dedifferentiated liposarcoma (DDLPS), was loaded in duplicate to observe the reproducibility between runs of the OGM technique. The results show a high consistency for the OGM technique (Supplementary Table S1).

#### 2.2.3. Detection of Genetic Aberrations

The analysis of the 33 cases by OGM resulted in an average of 408.4 alterations per sample (range: 22–2470) (Supplementary Table S2).

Expected alterations were identified in 97% (32/33) of the analyzed sarcomas: 11 with fusion genes, 10 with *MDM2* amplifications, and 11 with non-specific SVs and CNVs. The RVA pipeline detected alterations in 31/33 (94%) tumors (Table 3). These were determined based on routine diagnostic techniques. Both morphology and immunohistochemistry provide indications that suggest the presence of specific alterations. Additionally, all the fusions and CNV described in this study have primarily been investigated using FISH and a custom RNA-based NGS panel (Agilent, Santa Clara, CA, USA).

**Figure 2:**
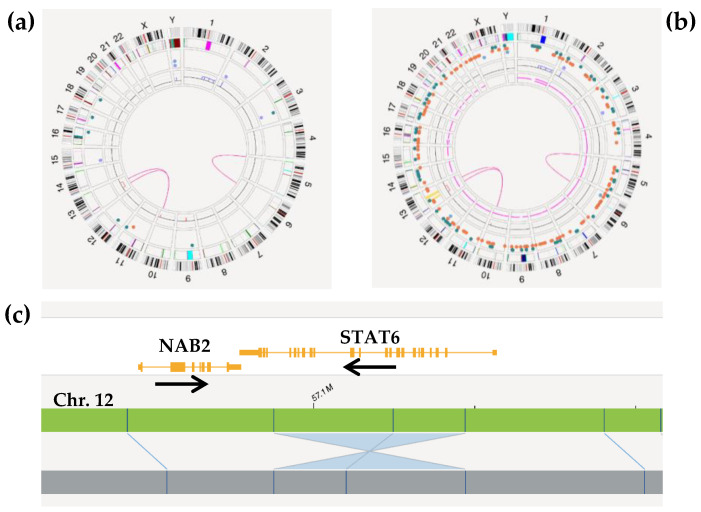
Optical genome mapping results of patient #36 (solitary fibrous tumor): (**a**) CircosPlot after the Rare Variant Analysis pipeline; (**b**) CircosPlot after De Novo Analysis, showing regions with loss of heterozygosity and a higher number of alterations; (**c**) view of the inv(12)(q13.3q13.3) inversion detected in the De Novo Analysis pipeline resulting in the *NAB2::STAT6* fusion gene. The black arrows indicate the direction of transcription of the genes.

De Novo Analysis, which identifies SVs > 500 bp, detected additional alterations in one tumor, corresponding to a solitary fibrous tumor with a *NAB2::STAT6* fusion (Figure 2). These genes are contiguous on chromosome 12 (12q13) but have opposite transcription directions, requiring a small inversion of a few kilobases to form the fusion gene [24,25]. The undetected case in OGM analysis was due to low percentage of neoplastic cells described in tenosynovial giant cell tumors (Table 3).

Supplementary Table S3 shows the CircosPlots, all fusion genes, and the annotations of the alterations found in each case analyzed by OGM according to the standards of the International System for Human Cytogenomic Nomenclature 2024 (ISCN) [26].

#### 2.2.4. Diagnosis Refinement

OGM analysis allowed a diagnosis refinement in 3/33 patients (9.1%). 

**Figure 3:**
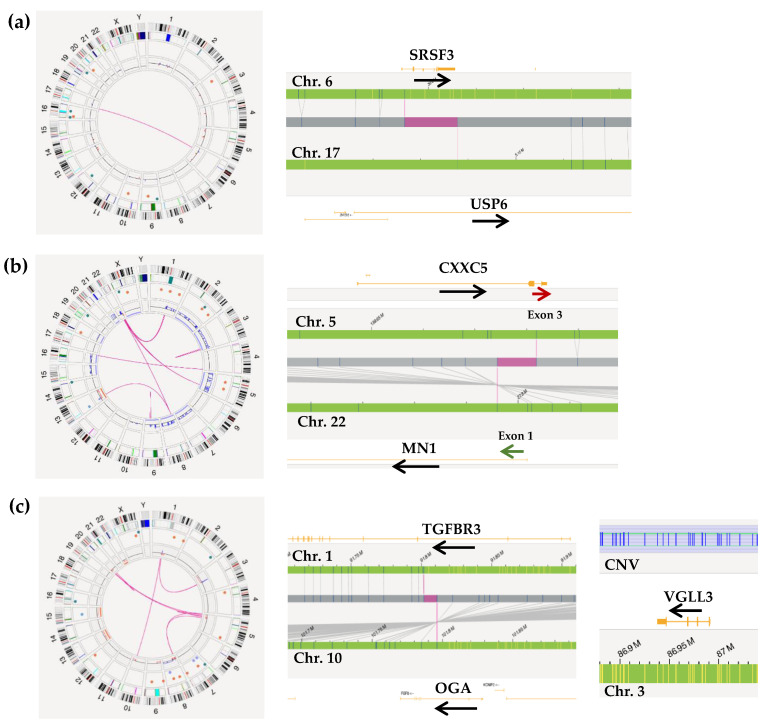
Optical genome mapping results in reclassified cases: (**a**) nodular fasciitis with t(6;17)(p21.31;p13.2)/*SRSF3::USP6*; (**b**) small round cell sarcoma with t(5;22)(q31.2;q12.1)/*MN1::CXXC5*, involving exon 1 of *MN1* (green arrow) and exon 3 of *CXXC5* (red arrow); (**c**) myxoinflammatory fibroblastic sarcoma with *VGLL3* amplification and t(1;10)(p22;q24)/*OGA::TGFBR3*. Chr: chromosome.

Patient #20, initially diagnosed with a low-grade myofibroblastic lesion, was reclassified as nodular fasciitis. OGM analysis detected the t(6;17)(p21.31;p13.2) translocation, confirmed by FISH, resulting in the *SRSF3::USP6* fusion gene, which is present in both aneurysmal bone cysts and nodular fasciitis (Figure 3a) [27].

Patient #30 was initially diagnosed with a small round cell sarcoma with Ewing-like morphology. However, OGM and FISH analysis did not detect any fusion typical of Ewing sarcomas. Nevertheless, OGM detected multiple translocations, among them the t(5;22)(q31.2;q12.1) translocation resulting in the *MN1::CXXC5* fusion gene. This translocation is typical of astroblastomas; cases of *MN1::BEND2* (also typical of astroblastomas) and *MN1::TAF3* fusions have begun to be described in soft tissue sarcomas [28,29]. This tumor has been classified as a small round cell sarcoma, opening the door for future research to more precisely determine its classification. (Figure 3b).

Patient #41, previously classified as a high-grade myxofibrosarcoma, was reclassified as a myxoinflammatory fibroblastic sarcoma, due to amplification of the *VGLL3*, monosomy of chromosome 13, and the t(1;10)(p22;q24) translocation, which results in the *OGA::TGFBR3* fusion gene, observed by OGM (Figure 3c) [30].

#### 2.2.5. CNV Aberrations

**Figure 4:**
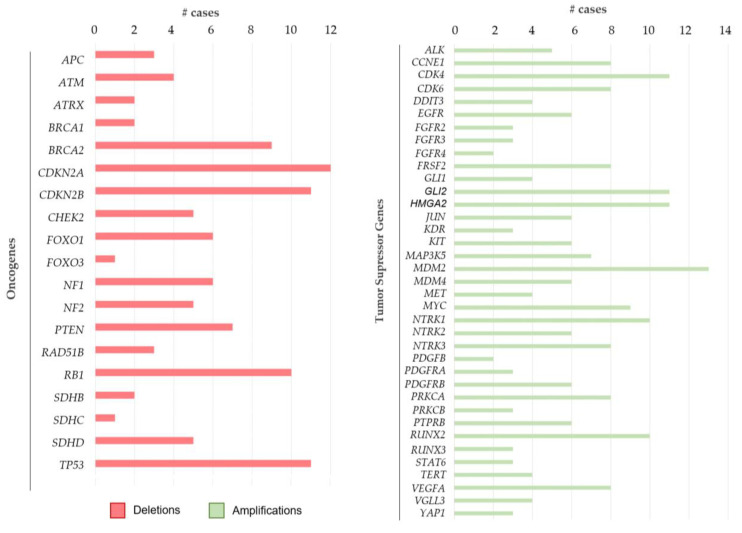
Number of alterations in oncogenes and tumor suppressor genes relevant in sarcomas detected by optical genome mapping in the 33 evaluable tumors. Tumor suppressor genes (**left**) are represented with red bars indicating deletions, while oncogenes (**right**) are shown with green bars indicating amplifications.

In addition to the defining alterations detected by OGM, amplifications in oncogenes and deletions in tumor suppressor genes of great importance in soft tissue and bone sarcomas were also detected (Figure 4 and Supplementary Table S4).

As expected, *MDM2* was the most amplified oncogene (13 cases, 39.4%), followed by *CDK4*, *GLI2,* and *HMGA2* (11 cases each, 33.3%), with co-amplifications in 5 cases (15%). *NTRK1* and *RUNX2* were also amplified in 10 cases (30.3% each).

Regarding tumor suppressor genes, the most deleted genes were *CDKN2A* (12 cases, 36.36%), *CDKN2B* (11 cases, 33.3%), *TP53* (11 cases, 33.3%), and *RB1* (10 cases, 30.3%).

#### 2.2.6. Complex Cases

Chromoanagenesis was detected in 17/33 patients (51.5%): 6 (35.3%) with chromoplexia, 10 (58.8%) with chromoplexia and chromothripsis, and 1 (5.9%) with chromothripsis. Furthermore, all dedifferentiated liposarcomas (DDLPSs), well-differentiated liposarcomas (WDLPSs), and atypical lipomatous tumors (ALTs) present chromothripsis in the long arm of chromosome 12, where amplified *CDK4*, *HMGA2,* and *MDM2* genes are localized.

## 3. Discussion

This study explores the use of OGM as an advanced tool to diagnose bone and soft tissue sarcomas, seeking to overcome the limitations of current techniques and adding information to predict behavior.

The principal limitation of this technique was the UHMW DNA extraction in adipocytic samples and myxofibrosarcomas, with a successful rate of around 65%. Challenges in extracting DNA in myxofibrosarcoma arise due to the low cellularity and myxoid matrix, while, in adipocytic tumors, it is due to the low nucleus-to-cytoplasm ratio and the presence of large lipid-rich cytoplasm in WDLPS, ALT, and lipomas. Consequently, using small tissue samples as the starting material makes UHMW DNA extraction particularly difficult. This issue can be mitigated by prioritizing tissue from surgical resections in lipomatous and myxofibrosarcoma tumors whenever available. Regarding DDLPSs, these tumors are composed of two morphologically distinct regions: WDLPS-like or ALT-like regions and dedifferentiated denser regions with a higher cell count where cytogenetic alterations responsible for the tumor’s increased aggressiveness are found. For optimal UHMW DNA extraction and tumor analysis, core biopsies must be performed in the latter region. On the other hand, patients with malignant tenosynovial giant cell tumors, defined by *COL6A3::CSF1* fusion and low percentage neoplastic cells, are better diagnosed by target PCR instead of OGM [31].

OGM identified defining alterations in 95% of tumors and, additionally, non-defining SVs and CNVs in 36%. Furthermore, it is important to highlight that the RVA pipeline was enough to find the expected alterations in 94% of tumors. Furthermore, in the OGM analysis workflow, we recommend first using the RVA pipeline, as it was enough to find the expected alterations in 94% of tumors. If the expected alterations are not identified, a De Novo Analysis is recommended, as it can detect smaller alterations.

OGM enhances the understanding of characteristic genomic alterations in soft tissue and bone sarcomas. In this context, our study highlights the utility of OGM, revealing amplifications in oncogenes—*MDM2* (39%), *CDK4* (33%), *HMGA2* (33%), and *GLI2* (33%)—as well as deletions in tumor suppressor genes—*CDKN2A* (36%), *CDKN2B* (33%), *TP53* (33%), and *RB1* (30%).

Regarding oncogenes, amplification of *MDM2* and *CDK4* is commonly observed in WDLPS, DDLPS, ALT, dedifferentiated parosteal osteosarcoma, and osteosarcoma NOS [32]. Previous studies have demonstrated that higher amplification levels of these genes are associated with poorer prognosis in DDLPS patients [33,34,35,36]. Additionally, for patients with DDLPS, targeted therapies inhibiting *MDM2*, such as milademetan and brigimadlin, are currently in phase 3 clinical trials (NCT04979442 and NCT06058793), while *CDK4/6* inhibitors, such as palbociclib, are already used in clinical practice [35,36]. However, in osteosarcomas, the clinical significance of these amplifications remains poorly understood. Otherwise, amplification of *HMGA2* has been reported in adipocytic tumors associated with a favorable prognosis, as it may contribute to tumor differentiation [37]. In fact, it can act as a prognostic marker in DDLPS, where the *MDM2/HMGA2* amplification ratio correlates with prognosis—lower ratio, better prognosis [34]. In addition, *GLI2* amplifications have been associated with osteosarcoma development and metastasis, making it a potential target for therapy in these tumors [38]. However, its role in WDLPS, DDLPS, and ALT is still unknown and could be interesting.

Referring to tumor suppressor genes, loss of *CDKN2A/B* has been reported to be associated with poor prognosis in soft tissue sarcomas [39]. More specifically, it has been described in Ewing sarcomas, osteosarcomas, and myxofibrosarcomas [40,41,42], being a potentially actionable gene for targeted therapies [43]. In addition, deletions, SVs, and LOH regions of *TP53* and *RB1* have also been identified in several sarcoma subtypes, particularly in leiomyosarcomas, myxofibrosarcomas, and undifferentiated pleomorphic sarcomas, where they play a significant role in tumor oncogenesis and progression [44,45]. Given the high percentage of patients with *TP53* losses (33%) detected by OGM, an extensive study is recommended to discard a possible Li–Fraumeni syndrome in young patients. However, despite their relevance, the impact of these tumor suppressor genes on patient prognosis and clinical management remains insufficiently explored.

Moreover, this study demonstrates that OGM provides a deeper understanding of the genomic complexity of soft tissue and bone sarcomas, detecting complex karyotypes with chromoanagenesis in 51.5% of patients. Regarding chromoplexy, rearrangement loops in fusion genes responsible for Ewing sarcomas have been reported as common in tumors with more aggressive behavior [46]; however, no cases have been detected in our cohort. Referring to chromothripsis, it is associated with more aggressive tumor behavior and poor prognosis in cancer patients [46,47,48,49,50]. In fact, Mandahl et al. [51] described chromothripsis-driven amplifications in the 12p regions, as well as in the 5p and 20q regions, in WDLPS and DDLPS, being more frequent in the latter, suggesting that it contributes to tumor aggressiveness. This aligns with our observations, as all patients with these tumors in our study presented chromothripsis in chromosome 12p and other chromosomes. Overall, further study of these complex events could lead to improved patient prognosis and even possible targeted therapies.

In addition, regarding osteosarcomas, a novel mechanism known as Loss-Translocation-Amplification (LTA) chromothripsis was recently described by Espejo Valle-Inclán et al. [50]. This process involves the loss of *TP53*, followed by translocation and amplification of various oncogenes, and appears to drive increased intratumoral heterogeneity and tumor clonal evolution [50]. Moreover, LOH regions have been identified as prognostic biomarkers for survival in osteosarcoma patients [50]. Although neither of these phenomena was observed in the two high-grade osteosarcoma cases in our study, both can be reliably detected using the OGM technique, enabling future research in this field.

In terms of time, OGM can generally be completed within 5–7 days, depending on sample quality and processing conditions, whereas NGS typically takes 7–14 days, especially when whole-exome or whole-genome sequencing is performed. Although targeted NGS panels provide faster results for known mutations, OGM offers a comprehensive, untargeted approach that enables the detection of a wider range of structural variations. This can be particularly valuable in rare cases or tumors with complex karyotypes, where traditional sequencing methods may fail to identify crucial alterations.

Despite its advantages, the OGM technique does come with certain limitations. One significant challenge is that formalin-fixed paraffin-embedded (FFPE) tissues often result in DNA that is unsuitable for this method. To facilitate the routine use of OGM in clinical laboratories, workflows would need to be adjusted to include the processing of fresh or appropriately frozen tissue. This necessitates that samples be frozen using liquid nitrogen and stored at −80 °C, which can present logistical challenges in many medical centers. Nevertheless, we strongly recommend the freezing of tumor biopsies for OGM studies, as this approach could substantially enhance prognostic accuracy and improve patient management. Moreover, an intrinsic limitation of OGM is its inability to detect point mutations, important in some sarcomas such as gastrointestinal stromal tumors (GISTs) or desmoid tumors [52,53]. To improve diagnosis, we propose the use of OGM together with NGS gene panels, achieving a more complete approach and better characterization of the patient’s tumor at the time of diagnosis.

## 4. Materials and Methods

### 4.1. Sample Processing and Selection

We selected biobanked samples from 53 adult patients, aged between 18 and 93 years old, diagnosed with soft tissue or bone sarcoma between 2022 and 2024 at our institution, a reference center, Hospital Universitari i Politècnic La Fe; which samples were preserved at the Biobanco La Fe.

The samples were sent fresh to the pathology lab and obtained by two different methods: core needle diagnostic biopsies percutaneously performed and surgical resections. Both types of samples were sent in an interval of no more than 30 min to prevent cold ischemia and DNA degradation. Hematoxylin and eosin (HE)-stained slides were prepared and reviewed by an expert pathologist to ensure the presence of at least 10% tumor cells in the tissue. The samples were weighed, frozen by immersion in liquid nitrogen for 3 min and stored in aliquots of 2 mL eppendorfs at −80 °C at Biobanco La Fe.

This study was approved by the Clinical Research Ethics Committee of the Hospital Universitari i Politècnic La Fe (No. 2023-984-1), and all patients signed an informed consent form in accordance with the recommendations of the Declaration of Human Rights, the Helsinki Conference, and institutional regulations.

### 4.2. Ultra-High Molecular Weight DNA Extraction and Labeling

For the OGM analysis, very long DNA molecules (>150 Kbp) are required. For this purpose, the Bionano Prep SP Tissue and Tumor DNA Isolation Extraction Kit was used according to the manufacturer’s instructions (Bionano Genomics, San Diego, CA, USA) [54], which allows the extraction of DNA molecules with a low degree of fragmentation due to the use of paramagnetic nanodiscs. After elution, UHMW DNA was homogenized and quantified using the Qubit Broad Range dsDNA Assay Kit, to ensure DNA concentration was between 50 and 150 ng/µL, as recommended by Bionano Genomics [54]. Subsequently, UHMW DNA labeling was performed using the Bionano Prep Direct Label and Stain Kit (Bionano Genomics) [55], which employs an enzymatic fluorescent labeling method targeting the CTTAAG motif with DLE-1 enzyme. This method does not introduce nicks in the gDNA, allowing the generation of long, contiguous genome maps (20–100 Mbp). Final quantification was performed using the Qubit High Sensitivity dsDNA Assay Kit (Thermo Fisher Scientific, Waltham, MA, USA) to ensure the DNA concentration was between 4 and 12 ng/µL, as recommended by Bionano Genomics.

### 4.3. Sample Loading on Chip, Reading by Saphyr, and Data Analysis

Samples labeled with concentrations between 4 and 12 ng/µL were loaded onto the Saphyr 3.3 chip (Bionano Genomics) and inserted into the Saphyr^®^ (Bionano Genomics) for imaging. UHMW DNA molecules were moved via electrophoresis, linearized, and imaged by a high-resolution camera. The images were converted into digital representations and compared to the fluorescently labeled human reference genome GRCh38 [56].

Results were obtained with quality parameters such as DNA collected (>150 Kbp), number of analyzed UHMW DNA strands (>150 Kbp and >20 Kbp), label density, mapping rate, genomic coverage, and positive (PLV) and negative (NLV) label variances. Aiming for 1500 Gbp of DNA, 300× coverage, and >70% mapping rate [17], samples were considered evaluable if the map rate was 40–50% with >150× coverage, or 50–70% with >100× coverage, based on our own experience analyzing solid tumors.

Data analysis was conducted using Bionano Access^®^ software (version 1.8.2), which consists of two bioinformatics analysis pipelines that were applied to all samples. First, Rare Variant Analysis (RVA), detects low VAF alterations, including SVs and CNVs larger than 5 Mbp by aligning DNA molecules >150 Kbp to the human reference genome and generating consensus maps. Secondly, samples were reanalyzed with the De Novo Analysis pipeline, designed to identify smaller SVs (<500 bp), differentiate homozygous from heterozygous alterations, and detect loss of heterozygosity (LOH) regions. This pipeline aligns molecules >200 Kbp and generates refined consensus maps for each allele [57]. Finally, a BED file containing 167 sarcoma-related genes was used for prioritization, focusing on SVs > 500 bp and CNVs > 500 bp.

### 4.4. Highly Complex Genomic Cases Analysis

High genomic complexity cases involve complex karyotypes with multiple inter- and intrachromosomal rearrangements, known as chromoanagenesis. First described in 2012 by Holland and Cleveland, chromoanagenesis refers to a catastrophic event involving multiple complex rearrangements in one or more chromosomal regions, encompassing processes like chromoplexy and chromothripsis [58].

- Chromoplexy: In the present study, chromoplexies are defined as chained multichromosomal rearrangements (≥3 chromosomes) with breakpoints less than five tags apart, unless deletion bridges were present [23,59,60].
- Chromothripsis: In this study, chromothripsis is determined as regions with copy number variations coexisting with more than seven chromosomal rearrangements in 50 Mb; furthermore, the rearrangements must be interspersed and uniformed in the affected region [61,62,63,64].

All chromoanagenesis events were manually inspected for validation.

## 5. Conclusions

Briefly, this study confirms the high utility of OGM as a diagnostic tool for soft tissue and bone sarcomas, identifying fusion genes, chromothripsis, chromoplexies, SVs, and CNVs, many of them affecting oncogenes and tumor suppressor genes. These findings open the door for future research focused on better understanding the mechanisms of sarcomas and developing targeted treatments.

## Figures and Tables

**Table 1:** General data of the studied population.

Age	(Years)
Average age	59
Age range	18–93
**Sex**	**n (%)**
Male	33 (62.3%)
Female	20 (37.7%)

**Table 2:** Histological nature of tumors and sarcoma subtypes.

Adipocytic	n (Total: 21)
Dedifferentiated Liposarcoma	7
Myxoid Liposarcoma	4
Atypical Lipomatous Tumor	4
Well-Differentiated Liposarcoma	3
Lipoma	2
Pleomorphic Liposarcoma	1
**Non-Adipocytic**	**n (Total: 15)**
Myxofibrosarcoma	11
Malignant Tenosynovial Giant Cell Tumor	1
Superficial Fibroblastic CD34+ Tumor	1
Solitary Fibrous Tumor	1
Low-Grade Myofibroblastic Lesion	1
**Bone**	**n (Total: 6)**
Malignant Giant Cell Tumor of the Bone	2
Osteosarcoma NOS *	1
Infantile Fibrosarcoma	1
Dedifferentiated Parosteal Osteosarcoma	1
Chondrosarcoma	1
**Uncertain Differentiation**	**n (Total: 7)**
Undifferentiated Pleomorphic Sarcoma	5
Monophasic Synovial Sarcoma	1
Atypical Fibromyxoid Ossifying Tumor	1
**Small Round Cell Sarcoma**	**n (Total: 4)**
Ewing Sarcoma	4

* NOS: Not otherwise specified.

**Table 3:** Comparison of genetic alterations detected in 33 assessable tumors by optical genome mapping with those expected in each case together with quality parameters.

Case	Soft Tissue or Bone Sarcoma Diagnosis	Expected Genetic Alteration	Detected by OGM	Total DNA > 150 Kb (Gbp)	Effective Coverage
3	G3 Undifferentiated Pleomorphic Sarcoma	CNV and SV	Yes	1513.96	366.74
5	G3 Myxofibrosarcoma	CNV and SV	Yes	1507.80	381.03
8	Myxoid Liposarcoma	***FUS::DDIT3***	Yes	640.20	107.44
9	Dedifferentiated Parosteal Osteosarcoma	***MDM2*** **amplification**	Yes	1500.01	379.76
12	G3 Undifferentiated Pleomorphic Sarcoma	CNV and SV	Yes	1502.11	351.86
14	G1 Myxofibrosarcoma	CNV and SV	Yes	1518.97	294.82
19	Dedifferentiated Liposarcoma	***MDM2*** **amplification**	Yes	1188.88	154.24
20	Low-Grade Myofibroblastic Lesion	CNV and SV *	Yes *	1511.15	202.35
22	Well-Differentiated Liposarcoma	***MDM2*** **amplification**	Yes	1502.11	391.16
23	G3 Myxofibrosarcoma	CNV and SV	Yes	1516.40	366.11
24	Atypical Lipomatous Liposarcoma	***MDM2*** **amplification**	Yes	1544.76	399.65
25	Lipoma	CNV and SV	Yes	1513.80	388.96
26	G3 Undifferentiated Pleomorphic Sarcoma	CNV and SV	Yes	1508.79	408.45
28	Dedifferentiated Liposarcoma	***MDM2*** **amplification**	Yes	1501.12	394.68
29	G3 Myxofibrosarcoma	CNV and SV	Yes	1456.12	317.03
30	Ewing Sarcoma	**Unknown rearrangement** *	Yes *	1545.00	417.77
31	Superficial CD34+ Fibroblastic Tumor	CNV and SV	Yes	1371.84	260.05
33	Malignant Tenosynovial Giant Cell Tumor	***COL6A3::CSF1***	No	1511.78	415.80
34	Atypical Fibromyxoid Ossifying Tumor	***PHF1*** **rearrangement**	Yes	1514.63	377.71
35	Dedifferentiated Liposarcoma	***MDM2*** **amplification**	Yes	1514.09	339.48
36	Solitary Fibrous Tumor	*NAB2::STAT6* **	Yes **	1522.03	402.56
39	Dedifferentiated Liposarcoma	***MDM2*** **amplification**	Yes	1548.60	413.73
40	G3 Myxofibrosarcoma	CNV and SV	Yes	1506.95	202.71
41	G3 Myxofibrosarcoma	CNV and SV *	Yes *	1501.94	379.13
42	Monophasic Synovial Sarcoma	***SS18::SSX1***	Yes	1518.48	344.09
43	G3 Osteosarcoma NOS	***MDM2*** **amplification**	Yes	1507.71	310.69
44	Ewing Sarcoma	***FUS::ERG***	Yes	724.03	122.79
46	High Grade Myxoid Liposarcoma	***FUS::DDIT3***	Yes	1453.70	415.64
47	G1 Myxofibrosarcoma	CNV and SV	Yes	1501.50	365.60
48	Ewing Sarcoma	***EWSR1::FLI1***	Yes	1502.46	394.20
49	Dedifferentiated Liposarcoma	***MDM2*** **amplification**	Yes	1515.32	358.94
50	Myxoid Liposarcoma	***FUS::DDIT3***	Yes	1516.61	364.82
51	Dedifferentiated Liposarcoma	***MDM2*** **amplification**	Yes	1501.60	185.60
Recommended values				>1500	>300

Bold text indicates defining alterations of a sarcoma subtype. * Diagnostic refinement after OGM analysis. ** Alteration detected by De Novo *Analysis*. CNV: copy number variation; Gbp: gigabase pair; G1: grade 1; G3: grade 3; LPS: liposarcoma; NOS: not otherwise specified; OGM: optical genome mapping; SV: structural variation.

## Data Availability

For inquiries about original data, please contact aberrub@upv.edu.es or mayordomo_emp@gva.es.

## Supplementary Materials

The following supporting information can be downloaded at: https://www.mdpi.com/article/10.3390/ijms26062820/s1.
